# Training-Stage Research Output as a Predictor of Long-Term Academic Productivity Among Orthopedic Sports Medicine Fellowship Faculty

**DOI:** 10.7759/cureus.113172

**Published:** 2026-07-22

**Authors:** William H Cusma, Conner Robbins, Suleiman Y Sudah, Yuri Han, Francis J Sirch, Andrew Miller, Alexander Rompala, Ryan Plyler

**Affiliations:** 1 Orthopedics, Monmouth Medical Center, Long Branch, USA; 2 Orthopedic Surgery, Massachusetts General Hospital, Harvard Medical School, Boston, USA; 3 Orthopedic Surgery, Robert Wood Johnson Medical School, Piscataway, USA; 4 Orthopedics, Rothman Orthopaedics, Philadelphia, USA; 5 Orthopedics, Shriners Children's Philadelphia, Philadelphia, USA

**Keywords:** academic productivity, fellowship, fellowship training, h-index, orthopedic sports medicine, orthopedic surgery, research, residency program, scholarly productivity

## Abstract

Background: The relationship between research productivity during training and subsequent academic output as a practicing orthopedic sports medicine surgeon remains poorly understood. Although prior studies have evaluated this association in other subspecialties, it has not been examined in orthopedic sports medicine. This study evaluated the correlation between academic productivity during each stage of training and future academic productivity. It also assessed whether the geographic region of training or practice impacts long-term research output. Materials and methods: The 2021-2022 American Orthopedic Society for Sports Medicine (AOSSM) directory was queried to identify fellowship faculty and associated institutions. Surgeons’ publications were counted across defined intervals: before residency, during residency, during fellowship, and after fellowship. Publication counts were compared by region of residency training and current practice location. A multivariable linear regression model identified predictors of the attending publication rate. Statistical significance was set at p < 0.05. Results: A total of 90 attending surgeons from 58 programs were included. The mean total publications were 97.6 ± 102, and the mean h-index was 26.3 ± 20.6. The mean attending publication rate was 5.00 ± 6.42 publications per year. Multivariable regression demonstrated that attending publication rate correlated with pre-residency (β = 0.327, p = 0.042), residency (β = 0.236, p = 0.001), and fellowship publications (β = 0.448, p < 0.001). No regional differences were observed based on residency training (p = 0.30). By practice location, the Northeast had the highest mean publication count, followed by the Midwest, West, and Southeast; differences were not significant (p > 0.29). Conclusions: Residency and fellowship publications significantly predicted long-term academic productivity, while pre-residency output showed a weaker association. Geographic location of training or practice did not significantly influence attending publication rate or h-index. These findings may inform evaluation of candidates for academic sports medicine positions.

## Introduction

Competition for orthopedic surgery residency positions is particularly intense. The match rate for orthopedic surgery residency applicants declined from 75% in 2016 to 66% in 2022 [1]. Repeat applicants have substantially lower match rates in recent years, driving an increased emphasis on differentiators among candidates, with many focusing on conducting research [2,3]. Due to the competitive nature of the orthopedic residency and fellowship application process, prospective applicants often strive to build strong academic credentials throughout their training, as residency and fellowship programs utilize research metrics as an adjunct to evaluate and rank applicants [4].

Consequently, the academic productivity of applicants to orthopedic surgery residencies has gained significant importance, with research output doubling between 2007 and 2014 [5]. This trend has been observed across multiple surgical specialties, with increasing numbers of publications among residency applicants in competitive fields [6,7]. The growing emphasis on research metrics in residency has raised questions about whether pre-residency research output reflects a genuine scholarly interest or strategic application enhancement [8]. Between 2000 and 2017, there was a self-reported increase in research activity among applicants [9]. The emphasis on research stems from the widely accepted belief that individuals who demonstrate strong academic productivity during their training years are more likely to make significant contributions to their field later in their careers and become prominent figures within the specialty. However, it remains unclear how research productivity during training is associated with future productivity as a practicing physician, and this relationship has not been specifically examined within orthopedic sports medicine. In the subspecialties of spine [10] and shoulder and elbow surgery [11], research output before and during training was associated with research productivity as an attending. Residency and fellowship publications showed stronger associations than pre-residency publications. Similar findings have been reported in other surgical subspecialties, including neurosurgery and plastic surgery, suggesting that training-stage research engagement may be associated with academic career trajectory across disciplines [12,13]. The h-index, originally proposed by Hirsch in 2005, has become a widely used metric for assessing research impact and has been applied to evaluate academic productivity across surgical disciplines, including orthopedic surgery [14-16].

For academic institutions seeking to hire physicians as well as orthopedic residency and fellowship programs evaluating trainees, it would be advantageous to understand how a trainee's research output relates to their future research productivity as a practicing physician. There is no published literature investigating the association of research conducted at different stages of training or the relationship of the geographic location of training institutions to the number of publications or h-index for the subspecialty of sports medicine. This information is particularly relevant given the differences in overall academic productivity across the different subspecialties of orthopedics [16]. Orthopedic sports medicine may differ from previously studied subspecialties in its training structure, fellowship culture, and balance between clinical and research demands, warranting subspecialty-specific investigation. This information could be valuable for trainees interested in pursuing a career in academic medicine, helping them establish appropriate research goals and select suitable training programs.

The purpose of this study was to evaluate whether research productivity before residency, during residency, and during fellowship is associated with long-term academic productivity among orthopedic sports medicine fellowship faculty and to assess whether geographic region of training or practice is associated with research output. We hypothesized that publications during later stages of training would show stronger associations with long-term academic productivity than pre-residency publications and that geographic location would not be significantly associated with research output. The primary questions of interest are (1) whether research output before residency, during residency, and during fellowship is associated with long-term academic productivity among orthopedic sports medicine fellowship faculty and (2) whether geographic region of training or practice is associated with research output.

## Materials and methods

We conducted a comprehensive review of all orthopedic sports medicine fellowship faculty members listed in the 2021-2022 American Orthopedic Society for Sports Medicine (AOSSM) fellowship directory. These faculty members typically assume significant roles in fellowship training and are often among the most academically accomplished individuals in the field. We collected data on each surgeon’s current practice location and educational background, including year of medical school graduation, residency, and fellowship. This information was obtained through online searches of the respective institutional websites. Surgeons were eligible for inclusion if they were featured in the AOSSM Sports Medicine Fellowship Directory and had complete documentation of their educational history. Surgeons were to be excluded if their educational background was unavailable through institutional websites, if their PubMed publication record could not be reliably identified due to name ambiguity, or if their Scopus profile was unavailable or unverifiable. None of the identified surgeons required exclusion, yielding a final cohort of 90. The absence of exclusions for name ambiguity reflects the ability to cross-reference publications with known institutional affiliations and subspecialty focus.

**Figure 1:**
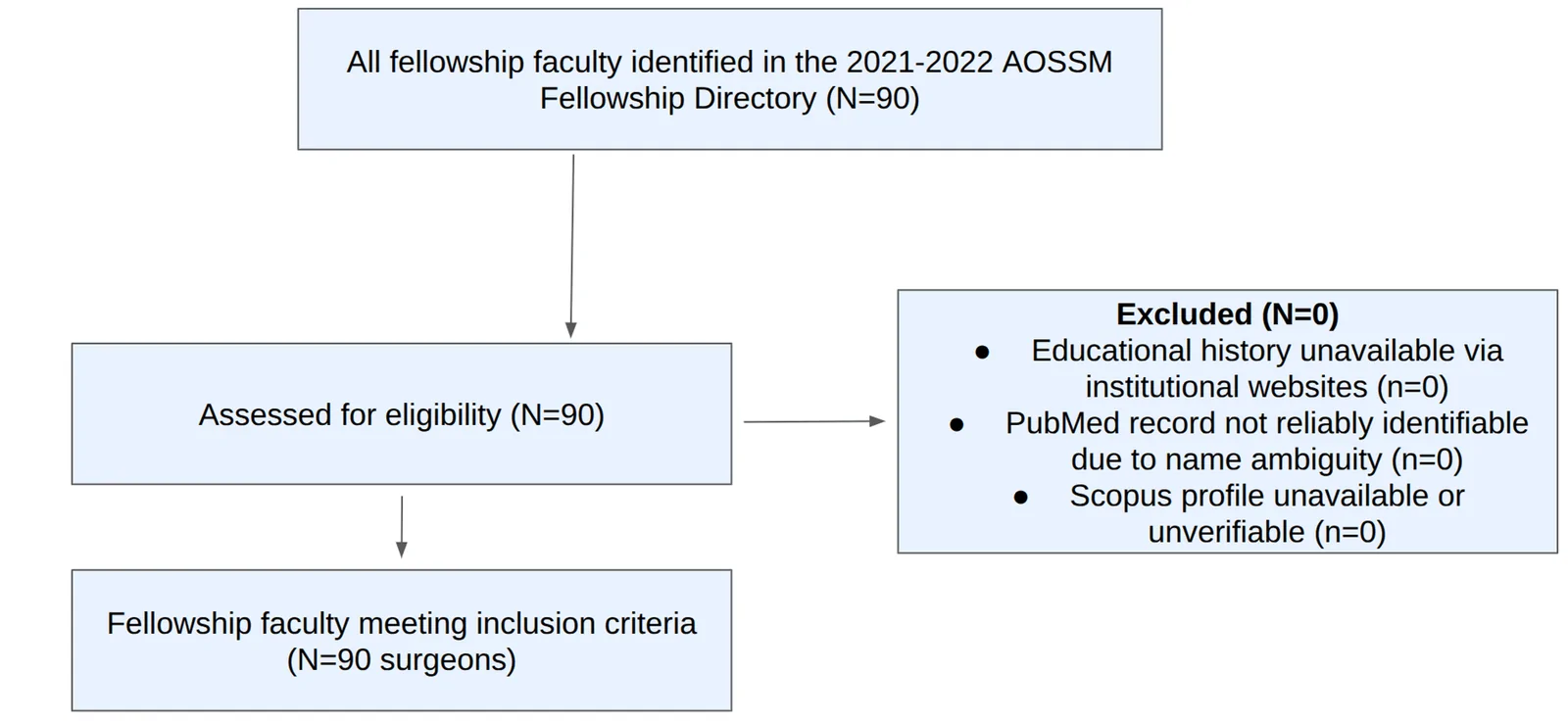
Flowchart of study participant identification and inclusion. A flowchart detailing the total number of fellowship faculty initially screened from the 2021-2022 AOSSM fellowship directory, specific reasons for exclusion, and the final included cohort AOSSM: American Orthopedic Society for Sports Medicine Image Credit: Authors using PowerPoint (Microsoft Corporation, Redmond, WA, USA)

A census-based sampling approach was used, in which all fellowship faculty listed in the 2021-2022 AOSSM Fellowship Directory were screened for eligibility. No formal sample size calculation was performed, as the study aimed to include the entire population of listed fellowship faculty meeting inclusion criteria. Of the total faculty initially screened, 90 surgeons from 58 programs met all inclusion criteria and were included in the final analysis (Figure 1).

For surgeons with non-traditional paths, training-stage windows were defined based on the recorded graduation dates for each stage. Publications were assigned to training stages based on publication date relative to these windows.

To compile information on each surgeon’s research output, we performed a PubMed search, recording the total number of publications up to July 1, 2023. The authors independently extracted publication data for each surgeon. Discrepancies in publication counts were resolved by consensus discussion. For each attending physician, we determined the number of articles published before residency, during residency, during fellowship, and after fellowship, based on their recorded medical education history. The attending publication rate was calculated by dividing the number of articles published after fellowship by the number of years since completing fellowship. Publication counts included all indexed publications regardless of authorship position.

Each surgeon’s h-index, a measure of research impact defined as the number of publications (h) that have each received at least h citations, was collected by examining each author’s Scopus profile as of July 1, 2023. [14] We reported descriptive statistics, including mean and standard deviation. Data were stratified based on the geographic location of residency training and current practice (Midwest, Northeast, Southeast, and West). The decade of fellowship graduation was also stratified. One-way analysis of variance (ANOVA) was used to compare continuous variables across groups. Multivariable linear regression was used to assess the association between publications at each training stage and attending publication rate. Both unstandardized (b) and standardized (β) regression coefficients were calculated. Standardized coefficients were derived using the formula β = b x (SD predictor/SD outcome) to allow comparison of the relative importance of associations across variables measured on different scales. Multicollinearity among predictor variables (pre-residency, residency, and fellowship publications) was assessed using variance inflation factors (VIFs), with values below 5 considered acceptable. Normality of residuals was evaluated using the Shapiro-Wilk test and inspection of the Q-Q plots. Homoscedasticity was assessed by plotting residuals against predicted values. This study received institutional review board exemption as it exclusively utilized publicly available data and did not collect any identifiable health information. All statistical analyses were performed using GraphPad Prism version 10.1.2 (GraphPad Software, Boston, MA, USA; www.graphpad.com). Statistical significance was defined as p < 0.05.

## Results

A total of 90 attending surgeons from 58 programs were included (Figure 1). The mean number of total career publications was 97.6 ± 102, with pre-residency publications at 0.73 ± 2.77, residency publications at 3.32 ± 8.10, fellowship publications at 2.48 ± 7.36, and post-fellowship publications at 90.5 ± 93.7 (mean rate: 5.00 ± 6.42 publications per year). The h-index was 26.3 ± 20.6 (Table 1).

**Table 1:** Summary data ^1 ^Data are presented as mean ± SD. Attending publication rate is defined as the number of publications after fellowship divided by the years since fellowship completion. h-index was obtained from Scopus profiles as of July 1, 2023. AOSSM: American Orthopedic Society for Sports Medicine, SD: standard deviation

Summary data	
Number of attendings	90
Number of programs	58
Total publications^1^	97.6 ± 102
Pre-residency publications^1^	0.73 ± 2.77
Residency publications^1^	3.32 ± 8.10
Fellowship publications^1^	2.48 ± 7.36
Attending publications^1^	90.5 ± 93.7
Attending publication rate^1^	5.00 ± 6.42
h-index^1^	26.3 ± 20.6

When stratified by decade of fellowship graduation, pre-residency publications did not differ significantly across cohorts. However, residency publications, fellowship publications, and the attending publication rate all increased significantly in more recent graduation cohorts. In the fellowship graduation cohorts of 1972-1990, 1991-2000, 2001-2010, and 2011-2017, the residency publications were 0.33, 1.41, 3.12, and 8.28, respectively (p = 0.020). Similarly, mean fellowship publications were 0.33, 0.59, 2.00, and 7.50, respectively (p = 0.009), and mean attending publication rates were 2.27, 3.31, 4.80, and 9.40, respectively (p = 0.005) (Table 2).

**Table 2:** Attending productivity measured against fellowship graduation period ^1^ Data are presented as the mean ± SD. p-values are calculated using one-way ANOVA. SD: standard deviation, ANOVA: analysis of variance

Attending start year	1972-1990	1991-2000	2001-2010	2011-2017	p-value
	N = 9 (10.0%)	N = 29 (32.2%)	N = 34 (37.8%)	N = 18 (20.0%)	
Pre-residency publications^1^	0.11 ± 0.33	0.17 ± 0.38	1.21 ± 4.34	1.06 ± 1.44	0.412
Residency publications^1^	0.33 ± 0.71	1.41 ± 2.67	3.12 ± 6.78	8.28 ± 14.3	0.020
Fellowship publications^1^	0.33 ± 0.50	0.59 ± 1.15	2.00 ± 3.40	7.50 ± 14.95	0.009
Attending publication rate^1^	2.27 ± 1.39	3.31 ± 3.12	4.80 ± 6.12	9.40 ± 9.82	0.005

**Figure 2:**
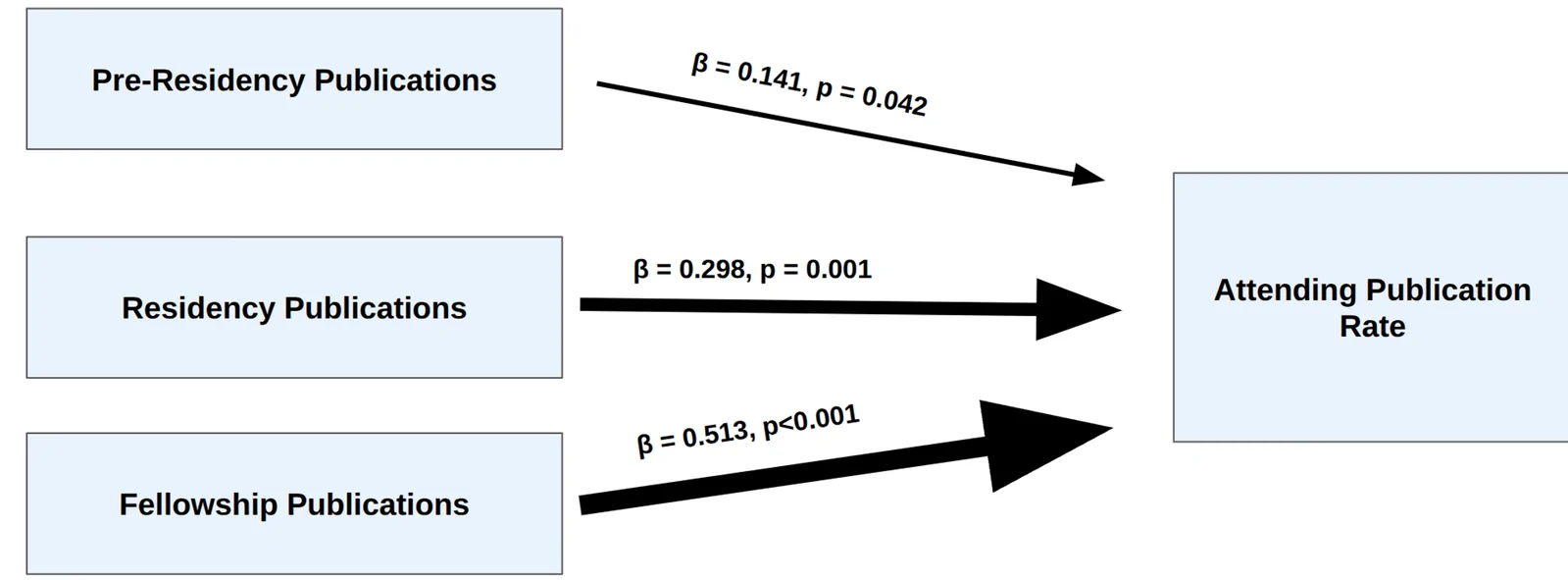
Illustrative schematic of the correlation between training-stage publication output and attending publication rate Image Credit: Authors using PowerPoint (Microsoft Corporation, Redmond, WA, USA)

In multivariable linear regression, attending publication rate was significantly associated with pre-residency publications (b = 0.327, β = 0.141, p = 0.042), residency publications (b = 0.236, β = 0.298, p = 0.001), and fellowship publications (b = 0.448, β = 0.513, p < 0.001). The standardized coefficients demonstrated that fellowship publications had the strongest relative association with attending publication rate, followed by residency and pre-residency publications. While the unstandardized coefficient for pre-residency publications (b = 0.327) was numerically larger than that for residency publications (b = 0.236), the limited variability in pre-residency publication counts (0.73 ± 2.77) results in a larger per-unit effect estimate; the standardized coefficient (β = 0.141) indicates that pre-residency publications had the weakest relative contribution to attending publication rate. However, h-index was not significantly associated with publications at any training stage: pre-residency (b = 0.741, β = 0.100, p = 0.383), residency (b = 0.426, β = 0.167, p = 0.252), or fellowship (b = 0.132, β = 0.047, p = 0.740) (Table 3; see Figure 2 for a visual schematic of association strengths). The standardized coefficients for h-index were uniformly small (β = 0.047-0.167) and non-significant (all p > 0.05), confirming that training-stage publication volume has minimal relative association with overall research impact.

**Table 3:** Multivariable linear regression of attending publication rate and h-index b = unstandardized regression coefficient, representing the change in the dependent variable per one-unit increase in the predictor. β = standardized regression coefficient, calculated as β = b × (SD predictor / SD outcome), allowing comparison of relative predictor importance across variables measured on different scales. P values were derived from multivariable linear regression models. Attending publication rate (SD = 6.42) and h-index (SD = 20.6) were modeled as separate dependent variables. Independent variables included pre-residency (SD = 2.77), residency (SD = 8.10), and fellowship (SD = 7.36) publication counts. SD = standard deviation

Model variables	Attending publication rate			h-index		
	Unstandardized coefficient (b)	Standardized coefficient (β)	p-value	Unstandardized coefficient (b)	Standardized coefficient (β)	p-value
Pre-residency publications	0.327	0.141	0.042	0.741	0.100	0.383
Residency publications	0.236	0.298	0.001	0.426	0.167	0.252
Fellowship publications	0.448	0.513	<0.001	0.132	0.047	0.740

When comparing productivity by region of residency, the Southeast had the highest mean number of publications at 127.0 ± 89.0, followed by the Northeast at 93.6 ± 85.7, the Midwest at 78.9 ± 125.0, and the West at 72.3 ± 67.7. Mean attending publication rates for the Southeast, Northeast, Midwest, and West were 6.46, 4.7, 4.94, and 4.72 publications per year, respectively. Regional comparisons did not reach statistical significance for attending publication rates; however, these analyses were underpowered given the small subgroup sizes, and the null results should be interpreted as inconclusive rather than evidence of a lack of influence of geography on productivity (Table 4).

**Table 4:** Analysis of publication characteristics based on the location of residency ^1^ Data are presented as the mean ± SD. p-values are calculated using one-way ANOVA. Northeast (n = 35, 38.9%), Midwest (n = 20, 22.2%), Southeast (n = 16, 17.8%), West (n = 17, 18.9%), and International (n = 2, 2.2%). SD: standard deviation, ANOVA: analysis of variance

Residency program location	Northeast	Midwest	Southeast	West	International	p-value
	N = 35 (38.9%)	N = 20 (22.2%)	N = 16 (17.8%)	N = 17 (18.9%)	N = 2 (2.2%)	
Attending publications^1^	93.6 ± 85.7	78.9 ± 125	127 ± 89.0	72.3 ± 67.7	11.5 ± 14.9	0.306
Attending publication rate^1^	4.70 ± 5.15	4.94 ± 8.44	6.46 ± 7.10	4.72 ± 6.02	0.88 ± 1.16	0.924
h-index^1^	28.5 ± 18.4	20.1 ± 23.7	35.6 ± 21.9	22.9 ± 17.6	4.50 ± 4.95	0.082

Based on current practice location, the Northeast had the highest mean publications (112.8 ± 89.4), followed by the Midwest (90.8 ± 123.0), the West (82.7 ± 88.7), and the Southeast (74.5 ± 66.3). Mean attending publication rates for the Northeast, Midwest, West, and Southeast were 5.2, 5.5, 4.3, and 4.9 publications per year, respectively. Mean h-index values for the Northeast, Midwest, West, and Southeast were 33.4, 23.5, 23.1, and 25.1, respectively. There was no statistically significant difference in attending publications, attending publication rate, or h-index by current practice location. However, these comparisons are similarly limited by small subgroup sizes and should be considered inconclusive (Table 5).

**Table 5:** Analysis of publication characteristics based on the location of current practice ^1^ Data are presented as the mean ± SD. p-values are calculated using one-way ANOVA. Northeast (n = 23, 25.6%), Midwest (n = 23, 25.6%), Southeast (n = 22, 24.4%), and West (n = 22, 24.4%). SD: standard deviation, ANOVA: analysis of variance

Attending practice location	Northeast	Midwest	Southeast	West	p-value
	N = 23 (25.6%)	N = 23 (25.6%)	N = 22 (24.4%)	N = 22 (24.4%)	
Attending publications^1^	112.8 ± 89.4	90.83 ± 123	74.5 ± 66.3	82.7 ± 88.7	0.558
Attending publication rate^1^	5.18 ± 5.41	5.52 ± 8.04	4.91 ± 7.31	4.30 ± 4.67	0.935
h-index^1^	33.4 ± 18.9	23.5 ± 24.0	25.1 ± 19.8	23.1 ± 19.0	0.294

## Discussion

In the increasingly competitive landscape of orthopedic training, research serves as a means of applicant differentiation and institutional recognition. Medical students often seek to distinguish themselves from other applicants in a crowded applicant pool, particularly following the transition of the United States Medical Licensing Examination (USMLE) Step 1 to pass/fail scoring [17]. Academic institutions value research productivity, as it enhances institutional reputation, improves grant funding, encourages industry partnerships, and positions faculty for roles in academic leadership [15,16]. In this study, we found that research productivity during residency and fellowship was significantly associated with attending publication rates among orthopedic sports medicine fellowship faculty. Pre-residency publication volume reached statistical significance but was comparatively weaker in its association. Our results are consistent with prior literature demonstrating that research output in residency and fellowship shows stronger associations with attending productivity [10,11,18]. Standardized regression coefficients demonstrated that fellowship publications had the strongest relative association with attending publication rate (β = 0.513), followed by residency (β = 0.298) and pre-residency publications (β = 0.141).

Publications are commonly used as an assessment tool for prospective applicants to residency and fellowship programs [4,19-21]. Since hiring and promotions in academia are strongly linked to academic productivity, it follows that programs prioritize applicants who demonstrate a propensity for academic productivity during training. These findings suggest that among current academic faculty, research conducted during fellowship shows the strongest association with long-term publication output, possibly due to focused mentorship, closer alignment with future career goals in academia, and self-selection of academically oriented trainees into research-intensive fellowship programs. However, these explanations remain speculative, as these constructs were not directly measured in this study. There was no significant association with the h-index. Standardized coefficients for h-index were uniformly small: residency publications had the largest relative effect (β = 0.167), followed by pre-residency (β = 0.100) and fellowship publications (β = 0.047), none of which reached statistical significance. This suggests that while publication quantity during training is associated with future output, it does not necessarily correlate with research impact as measured by h-index. This dissociation between publication volume and h-index may reflect the time required to accumulate citations, as well as the multifactorial nature of research impact, which depends on study quality, journal prestige, and topic relevance rather than quantity alone [14,15].

One potential explanation for the association between residency and fellowship publications and attending productivity is that residents and fellows are more likely to engage in research if they are genuinely interested in research and, therefore, more likely to pursue an academic career. Furthermore, programs that emphasize research are likely looking for high-output applicants for fellowship and faculty positions. This differs from medical school, where participation in research may be more driven by the desire to enhance one's application to secure a residency position.

With the transition of USMLE Step 1 to pass/fail scoring, applicants are likely to prioritize research to demonstrate their interest in a field [17,21]. High-achieving students who would have previously dedicated significant time to board examination preparation may now redirect those efforts toward research. Consequently, it is plausible that future applicants will have more research experience on their applications, and the quantity of these experiences may become a key metric for application comparisons. However, our finding that pre-residency publications had the weakest standardized association (β = 0.141) with long-term productivity suggests that programs should interpret pre-residency research output cautiously, as it may reflect strategic application-building rather than genuine academic interest.

The increasing emphasis on research productivity during training also raises important questions about academic resource equity and access. Trainees at institutions with greater research infrastructure, dedicated research time, and established mentorship networks may have inherent advantages in accumulating publications, independent of individual aptitude or interest [22,23]. This institutional effect has been documented in other surgical specialties, where trainees at high-volume academic centers consistently outperform peers at community-based programs in publication output [24,25]. Future studies should examine whether institutional research resources moderate the association between training-stage publications and long-term productivity.

Our study also revealed a trend of increasing publication numbers among more recent graduation cohorts, aligning with previous studies suggesting that research expectations and output have increased over time. Specifically, attending publication rates increased from 2.27 publications per year for those graduating between 1972 and 1990 to 9.40 publications per year for those graduating between 2011 and 2017 (p = 0.005). This trend likely reflects the growing emphasis on research in residency and fellowship training, increased availability of research resources, and the expanding role of academic productivity in academic medicine careers.

The regional analyses were underpowered and should be interpreted as inconclusive. While the Northeast had the highest mean number of publications (112.8 ± 89.4) and h-index (33.4), these differences did not reach statistical significance, likely due to the small sample sizes within each regional subgroup. The p = 0.082 finding for h-index by residency region is suggestive and should not be interpreted as evidence of no association; rather, it warrants further investigation with adequately powered samples. The concentration of large academic medical centers in the Northeast may contribute to the observed trends, though definitive conclusions cannot be drawn from the current data.

These findings have several implications for the orthopedic sports medicine specialty. For trainees, our data suggest that sustained research engagement during residency and fellowship, rather than pre-residency research alone, shows the strongest association with long-term publication output among those who ultimately become academic faculty. For fellowship programs and academic institutions, residency and fellowship publication counts are associated with attending publication rates within this cohort. However, these associations have not been validated prospectively and should be considered alongside other factors such as research quality, authorship position, and mentorship. Publication counts should not be used as a surrogate measure for research quality or impact when evaluating candidates for faculty positions. For residency and fellowship program directors, these findings underscore the importance of establishing research environments that support trainee productivity, as early-career scholarship appears to be associated with patterns that persist into independent practice among surgeons who remain in academic practice.

This study has several limitations that warrant consideration. Most fundamentally, the cohort is restricted to surgeons who are already academic faculty, which represents selection based on the outcome and limits the ability to draw predictive conclusions. Without a comparison group of trainees who published during training but did not enter or sustain academic practice, the observed associations should be interpreted as descriptive rather than predictive. Additionally, the results communicate associations and do not establish causation; unmeasured confounders include mentorship quality, institutional resources, protected research time, grant funding, and individual career preferences, which may account for both early and later publication productivity.

Several analytical considerations also merit acknowledgment. The attending publication rate outcome is overdispersed (SD > mean), which raises concerns about the appropriateness of linear regression; count-based models such as negative binomial or Poisson regression with an offset for years since fellowship may be more suitable. Graduation era, a likely confounder of both predictors and outcome given the significant secular trends observed across cohorts, was not included as a covariate, and its omission may inflate the magnitude of training-stage associations. The pre-residency predictor shows minimal variability and may be influenced by outliers, rendering its marginal statistical significance fragile. Furthermore, the three training-stage predictors are inherently collinear, and the relative ordering of their standardized coefficients may be unstable at this sample size.

Methodological limitations related to data collection should also be noted. Publication counts were drawn from PubMed while h-index values were drawn from Scopus, and differences in database coverage may partly explain the dissociation between attending publication rate and h-index findings. Assigning publications to training stages by publication date may systematically misclassify lagged fellowship-era papers into the attending window, and publication counts assign equal weight to all authorship positions, which may favor members of large collaborative teams. The use of PubMed as the sole source for publication counts may have underestimated total research output, as non-indexed journals, book chapters, and conference proceedings were not captured. The regional analyses were underpowered, particularly the international subgroup (n = 2), and their null results should be considered inconclusive rather than evidence that geography does not influence productivity. The h-index is not specific to sports medicine, and the study population was limited to fellowship faculty listed in the AOSSM directory, which may introduce selection bias toward more academically productive surgeons and limit generalizability to those in private practice.

Future studies should address these limitations through several approaches. Prospective longitudinal designs tracking research productivity from medical school through independent practice, with inclusion of a comparison group of trainees who do not enter academic careers, would enable genuine predictive validation. Reanalysis using count-based regression models with graduation year as a covariate, bootstrapped confidence intervals on standardized coefficients, and first/last-author subanalyses would address the primary analytical limitations of the current work. Examination of research quality metrics, such as journal impact factor and citation counts of individual publications, alongside quantity would provide a more nuanced understanding of academic trajectory. Publication-date lag sensitivity analyses and curriculum vitae/open researcher and contributor ID (CV/ORCID) validation of a subsample would strengthen the reliability of training-stage classification. Finally, investigation of research productivity among sports medicine surgeons in non-academic and community practice settings would broaden the applicability of these findings.

## Conclusions

Research output during residency and fellowship was significantly associated with publication rate among attending orthopedic sports medicine fellowship faculty. Pre-residency publications were also associated with attending publication rates, though this association was weaker and should be interpreted cautiously given the limited variance in pre-residency publication counts. Standardized regression coefficients demonstrated that fellowship publications had the strongest relative association, followed by residency and pre-residency publications. Training-stage publications were not significantly associated with h-index, suggesting that publication volume during training does not necessarily correspond to research impact as measured by citation-based metrics. Regional analyses were inconclusive due to small subgroup sizes. These findings are descriptive associations within a cohort of established academic faculty and should not be interpreted as predictive of academic career outcomes without prospective validation. Residency and fellowship publication counts may be considered alongside other factors when evaluating candidates for academic sports medicine positions, though their independent value for this purpose remains to be established.
